# Ruptured Thoracolumbar Perimedullary Arteriovenous Fistula during Pregnancy Complicated by Cerebral Subarachnoid Hemorrhage and Brainstem Hematoma: A Case Report

**DOI:** 10.3390/brainsci10080561

**Published:** 2020-08-15

**Authors:** Jan Sroubek, Ladislava Janouskova, Jan Klener

**Affiliations:** 1 Department of Neurosurgery, Na Homolce Hospital, Roentgenova 5, 150 30 Prague, Czech Republic; jan.klener@homolka.cz; 2 Department of Radiology, Na Homolce Hospital, Roentgenova 5, 150 30 Prague, Czech Republic; ladislava.janouskova@homolka.cz

**Keywords:** perimedullary arteriovenous fistula, subarachnoid hemorrhage, brainstem hematoma, thoracolumbar, pregnancy

## Abstract

Intradural spinal arteriovenous fistulas (sAVF) are spinal vascular lesions that usually manifest due to myelopathy or local symptoms caused by venous congestion and ischemia. In addition, perimedullary arteriovenous fistulas (PMAVF) in particular may rupture and cause subarachnoid or intramedullary hemorrhage along with relevant symptoms. Subarachnoid hemorrhage (SAH) can propagate into cranial space with clinically dominant symptoms and signs of typical aneurysmal intracranial SAH. The standard workup for cerebral SAH, after excluding an intracranial source of hemorrhage, is usually limited to a cervical spine MRI; therefore, thoracolumbar sources of hemorrhage can be missed, or their diagnosis may be delayed. Here we present a case of a pregnant patient who presented with cerebral SAH. The source of hemorrhage was not initially identified, leading to a presumptive diagnosis of benign pretruncal non-aneurysmal SAH. The correct diagnosis of spinal thoracolumbar PMAVF was revealed 2.5 months later due to the progression of local symptoms. While the diagnosis was being refined and endovascular treatment was being planned (but delayed due to pregnancy), there was a recurrence of intraconal hemorrhage followed by brainstem hemorrhage. This led to significant clinical deterioration. The PMAVF was then treated microsurgically and the patient experienced partial recovery.

## 1. Introduction

Intradural spinal arteriovenous fistulas (sAVF) are rare vascular lesions that usually present with progressive pain, radiculopathy, and myelopathy as a consequence of venous congestion. Rarely, intramedullary hemorrhage or subarachnoid hemorrhage (SAH) may occur as well [1]. Magnetic resonance imaging (MRI) of the spine and spinal angiography may elucidate the type of lesion and the proper treatment modality can be selected [2,3,4]. However, the initial clinical manifestation can be nonspecific and may mislead the initial diagnosis; the exact diagnosis may become apparent only after more severe and specific clinical manifestation transpire [5]. 

Here, we present a case of a patient, in whom sAVF manifested during pregnancy; the initial presentation was cranial SAH. The secondary presentation occurred in the postpartum period (just as the diagnosis of thoracolumbar perimedullary AVF was made): first as intramedullary hemorrhage and subsequently as brainstem hemorrhage accompanied by neurological deterioration.

## 2. Case Report

**Figure 1:**
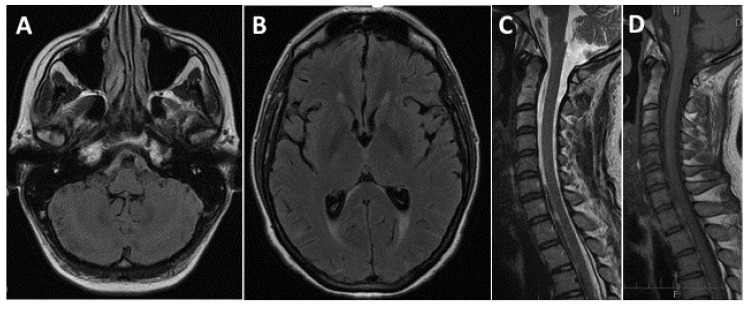
Initial MRI, subarachnoid hemorrhage. (**A**) Axial FLAIR image, premedullary subarachnoid hemorrhage (SAH). (**B**) Axial FLAIR image, SAH on the posterior convexity. (**C**) Sagittal T2—weighted image, premedullary SAH. (**D**) Sagittal T1—weighted image, premedullary SAH.

A 37-year-old pregnant (20th week), otherwise healthy female, reported a history of chronic low back pain for many years. During pregnancy, she noted some worsening of this back pain, albeit she was still able to easily control her symptoms with occasional use of paracetamol (acetaminophen). On the day of her initial presentation she awoke disoriented and with memory loss (as is typical for transient global amnesia). She enjoyed full memory recovery within several hours but complained of a marked but tolerable headache. She was examined by a local neurologist and was discharged home. Two days later she was admitted to a tertiary-care hospital because of symptom progression. On admission, the patient was suffering from cephalea, nausea, and had meningeal signs. MRI of the brain and cervical spine showed SAH in the prepontine and premedullary cisterns and on the convexity. No vascular abnormalities in the craniocervical junction or the cervical area could be identified on the MRI (Figure 1A–D). Digital subtraction angiography of the cranial vessels did not reveal any aneurysms.

**Figure 2:**
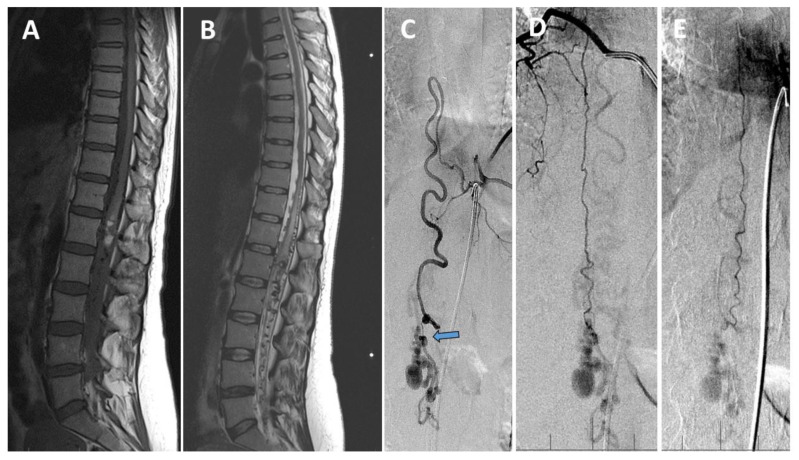
Intramedullary hematoma and perimedullary arteriovenous fistula (PMAVF). (**A**) Sagittal T1—weighted MRI showed an intraconal hemorrhage at the level of L1. (**B**) Sagittal T2—weighted MRI demonstrated myelopathy and dilated veins at the level of T10 to L5. (**C**–**E**) Initial spinal digital subtraction angiography revealed supplying arteries of the PMAVF; (**C**) Dilated anterior spinal artery with the increased flow is the main PMAVF feeder (blue arrow pointing to the fistula). (**D**) Right posterior spinal artery and (**E**) left posterior spinal artery also supplying the PMAVF.

As the symptoms diminished, she was discharged home with the presumptive diagnosis of pretruncal non-aneurysmal SAH. After a period of 2.5 months and in her 32nd week of pregnancy, she underwent an MRI examination for moderate back pain. This identified a spinal vascular lesion with a large ectatic venous aneurysm at the level of L1. Dilated perimedullary veins could be seen in the T10 to L5 range. Cesarean section was recommended, leading to the delivery of a healthy child in the 33rd week of pregnancy. Two days later, as she was recovering in the obstetric ward, the patient developed progressive lumbar pain and lower limb weakness along with partial urinary incontinence. A repeat MRI examination revealed a conus medullaris hemorrhage at the L1 level, accompanied by thoracic myelopathy from T10 inferiorly (Figure 2A,B). The patient was admitted to our hospital and spinal angiography identified a perimedullary AVF (PMAVF) type B centered at the L1 level, supplied by a pedicle from the dilated anterior spinal artery (ASA) (Figure 2C) and by smaller pedicles from both posterior spinal arteries (PSA) (Figure 2D,E). A large venous aneurysm was also identified. 

**Figure 3:**
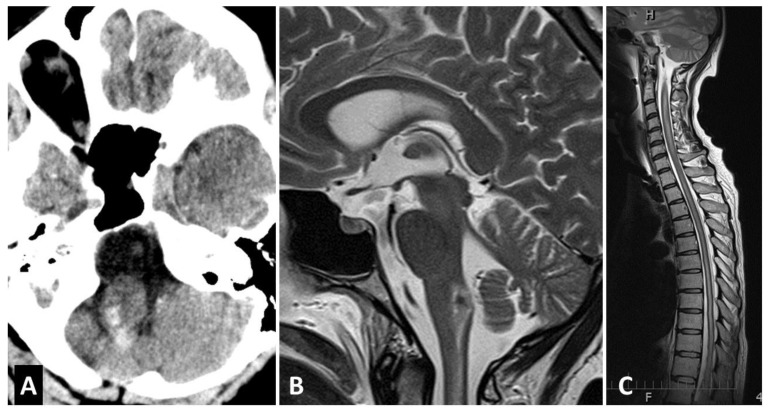
Brainstem hemorrhage. (**A**) Axial CT showed brainstem hemorrhage, (**B**,**C**) Sagittal T2—weighted MRI confirmed brainstem hemorrhage and revealed cervical and thoracic myelopathy.

The patient made a near-complete spontaneous functional recovery within several days. Microsurgical treatment was considered to be of higher risk compared to endovascular intervention. Therefore, transfer to a specialized endovascular center was scheduled. However, before her transfer during a bowel movement, she rapidly developed quadriparesis, bulbar syndrome, and dyspnea, requiring urgent intubation. Computed tomography (CT) showed a brainstem hemorrhage (Figure 3A,B) and extensive cervical and thoracic myelopathy appeared on MRI (Figure 3C). 

**Figure 4:**
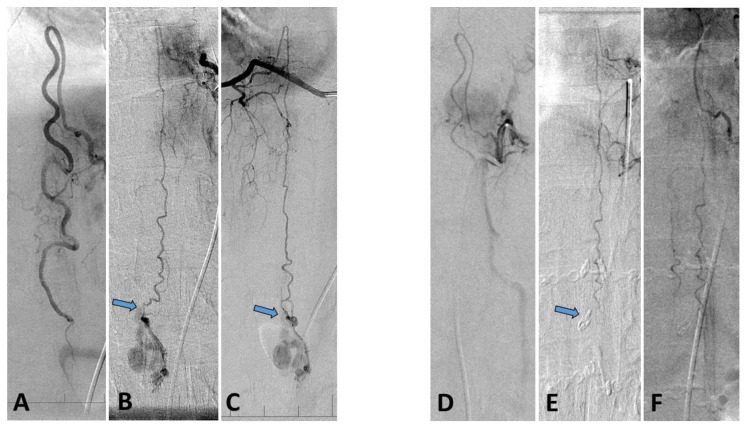
Spinal digital subtraction angiography after neurological deterioration. (**A**–**C**) Preoperative and (**D**–**F**) postoperative findings. (**A**) Spontaneous occlusion of the pedicle feeding the PMAVF from the dilated anterior spinal artery (ASA). (**B**) Left posterior spinal artery (LPSA) supplying the PMAVF (arrow—point of shunting). (**C**) Right posterior spinal artery (RPSA) supplying the AVF (arrow—point of shunting). (**D**) Normalized flow in the ASA after surgical fistula occlusion. (**E**) LPSA after surgery, note the clip at the fistula location (arrow). (**F**) RPSA after surgery.

Thoracolumbar MRI did not reveal any change in the intraconal hemorrhage. Second spinal angiography to close the fistula was attempted but the AVF feeding pedicle from the ASA was found to have spontaneously occluded. Both PSA (that were still supplying the PMAVF) were not amenable to percutaneous intervention (Figure 4A–C). Neurosurgical intervention was postponed due to the critical state of the patient. Within 3 days the patient was extubated and her brainstem function recovered. However, the patient had severe paresis of the right hand and lower limb paraplegia accompanied by urinary incontinence and decreased sensation under the level of C7. 

A multidisciplinary team recommended surgical revision as the last-line treatment in hopes of preventing further, potentially fatal hemorrhage. During surgery, subdural and intraconal hemorrhage were visualized and were partially evacuated. A fistula was identified and obliterated. There was an arterialized venous aneurysm distal to the fistula, which was found to be the source of the hemorrhage.

Over the course of the following year, the patient’s neurological deficit improved slightly, allowing her to ambulate with a walker (Aminoff 4). However, her urinary incontinence did not improve, nor was there any improvement in her diminished sensation from the T10 level inferiorly. Postoperative spinal angiography revealed complete obliteration of the vascular lesion with no residual filling (Figure 4D–F) An MRI showed improvement with respect to myelopathy. 

## 3. Discussion

Several classification schemes have been proposed to facilitate better understanding and management of spinal AVMs and AVFs. The most widely used classification divides these lesions into four types [6]. Type I are spinal dural AVFs, type II are intramedullary AVMs, type III are juvenile or metameric lesions, and type IV are perimedullary AVFs. A more recent classification proposed by Spetzler et al. [1] recognized two additional types: Extradural spinal AVFs and conus medullaris AVMs. The perimedullary type of AVF (PMAVF), a case of which is reported here, can be subdivided according to the feeder vessel, AVF size and drainage into three subtypes (A,B,C) and the type of treatment can be individualized as discussed below further [1,2,4].

PMAVF are difficult to diagnose because the symptoms related to venous hypertension and medullary hypoxia may be non-specific. Nonetheless, PMAVF may ultimately result in severe disability. When the first symptoms appear, there is a 50% probability of progression to a bed-ridden state (Aminoff 5) within 3 years [7]. Therefore, timely recognition of the sAVF is essential to allow therapy to meet a narrow window of opportunity [3]. Less frequently, the symptoms of PMAVF may be caused by rupture and hemorrhage either in the intramedullary or in the subarachnoid space [3,4]. 

There are rare reports of these lesions presenting as cranial SAH with minimal myelopathic complaints, directing the diagnostic protocol onto a false pathway [8], with the correct diagnosis relegated to the bottom of the differential. When intracranial aneurysm or other conceivable sources of SAH are not identified, MRI of the cervical spine should be performed to identify a possible lesion. If there are no anomalies on cervical MRI, the thoracolumbar area should be considered as a rare but possible source of SAH. Aside from vascular malformations [9], other pathologies of the thoracolumbar region may rarely manifest as SAH and need to be considered (e.g., myxopapillary ependymoma [10], spinal schwannoma [11]). 

In recent literature reviews [8,12], there were reports of only a handful of thoracolumbar AVFs that manifested purely with cranial SAH. Most of these were correctly diagnosed before the onset of neurological deterioration [13,14,15,16]. A few instances resembled the case presented herein in that the correct diagnosis was revealed only after a second hemorrhage and clinical deterioration. The first was a case of a 14-month-old child with cranial SAH and hemocephalus with no symptoms attributable to the spine area. Three days after symptom onset after the onset of paraparesis, thoracolumbar PMAVF was identified, the intramedullary hematoma was evacuated surgically and the fistula treated endovascularly [17]. Another case was a 28-year-old male with cranial SAH, initially also free of spinal symptoms. He became paraparetic 7 days after the occurrence of cranial SAH. An AVF at the T9 level was identified and treated surgically, leading to a fairly good clinical outcome [18]. The origin of SAH in the above-mentioned cases, as well as in our case, was very likely caused by the rupture of pathological AVF vessels with little or no local symptomatology. Manifestations of cranial SAH dominated the clinical picture, although the blood had to spread from the thoracolumbar area into the cranium. 

None of the above mentioned cranial SAH with clinically silent thoracolumbar sAVF manifested during pregnancy. It has been postulated that physiological hormonal changes associated with pregnancy, along with compression of venous outflow and augmented intravascular volume make the spinal malformation particularly susceptible to clinical manifestations due to vessel rupture or venous congestion [19]. The risk of clinical deterioration decreases after delivery and some spinal AVFs may even spontaneously regress [20,21]. In our case, the PMAVF ruptured in the middle of pregnancy but due to the lack of local or myelopathic symptoms, a correct diagnosis was not established at the time of the initial presentation. Chronic, mild lower back pain was not thought to be relevant and was thus not linked to the cranial SAH. Instead, the correct diagnosis was established 2.5 months later owing to a routine MRI of the thoracolumbar region (indicated in the setting of ongoing mild lower back pain). Because the fetus was mature at that time and because the patient had minimal local symptoms, a decision was made to first proceed to infant delivery. Subsequent deteriorating intramedullary hemorrhage and brainstem hemorrhage occurred after childbirth, when the intrabdominal pressure should theoretically have already normalized [22].

Brainstem or cerebellar hemorrhage can rarely appear as a result of an AVF in the craniocervical region [23]. In the case of thoracolumbar AVF, brainstem or cerebellar hemorrhage is a very unique and exceptional manifestation; to the best of our knowledge, this has not yet been reported in the literature. We hypothesize that increased intra-abdominal pressure raised the pressure in the perimedullary veins, which was transmitted to the level of the craniocervical junction and led to hemorrhagic venous infarction. Another possible mechanism of pathogenesis invokes the rupture of an ectatic vein related to the AVF. However, no supporting evidence for this mechanism was seen on MRI or angiography.

Treatment of this condition is individualized and requires a thorough understanding of the AVF angioarchitecture. Detailed spinal angiography is required and all possible supplying vessels must be identified. In general, the treatment of choice depends on the type of perimedullary AVF. Type A (small size, one feeding artery, generally the ASA) is usually a good candidate for direct clipping of the fistula, whereas type B (intermediate size, major feeder from the ASA) is usually treated by combined endovascular techniques and surgery. Type C AVFs (giant size, many feeding pedicles usually from a dilated ASA) are treated endovascularly [1,2]. In our patient, initial spinal angiography identified a type B perimedullary AVF supplied by a dilated ASA and both PSA. The risk of surgery and endovascular treatment was assessed and the endovascular treatment in a specialized center was eventually requested. This transfer had to be canceled following another hemorrhage leading to a patient’s neurologic deterioration. Later, microsurgical AVF extirpation was indicated and successfully performed. 

## 4. Conclusions

Perimedullary AVF is a rare disease, often associated with nonspecific symptoms but leading to a malignant course if not treated promptly. As shown in this report, not only cervical but also thoracolumbar pathology should be considered if SAH occurs and an intracranial source of hemorrhage is not revealed. Early diagnosis and treatment in this particular case may have avoided intramedullary and brainstem hemorrhages causing clinical deterioration.
